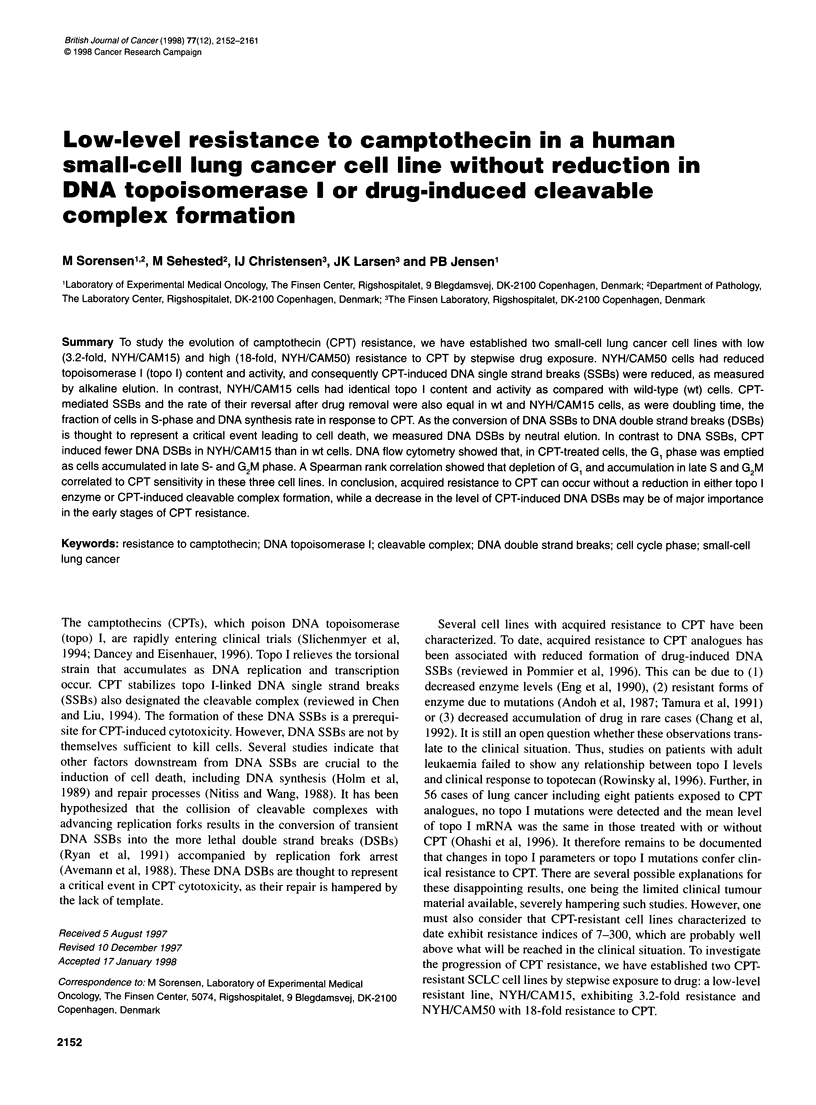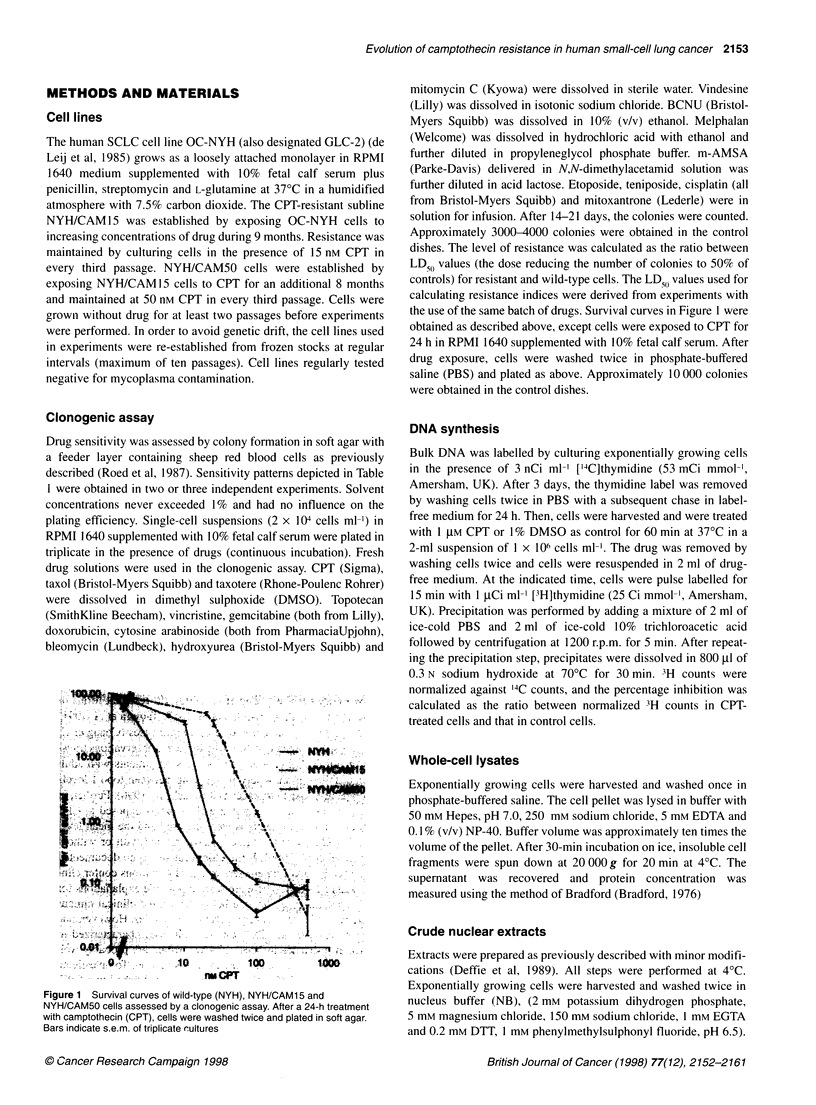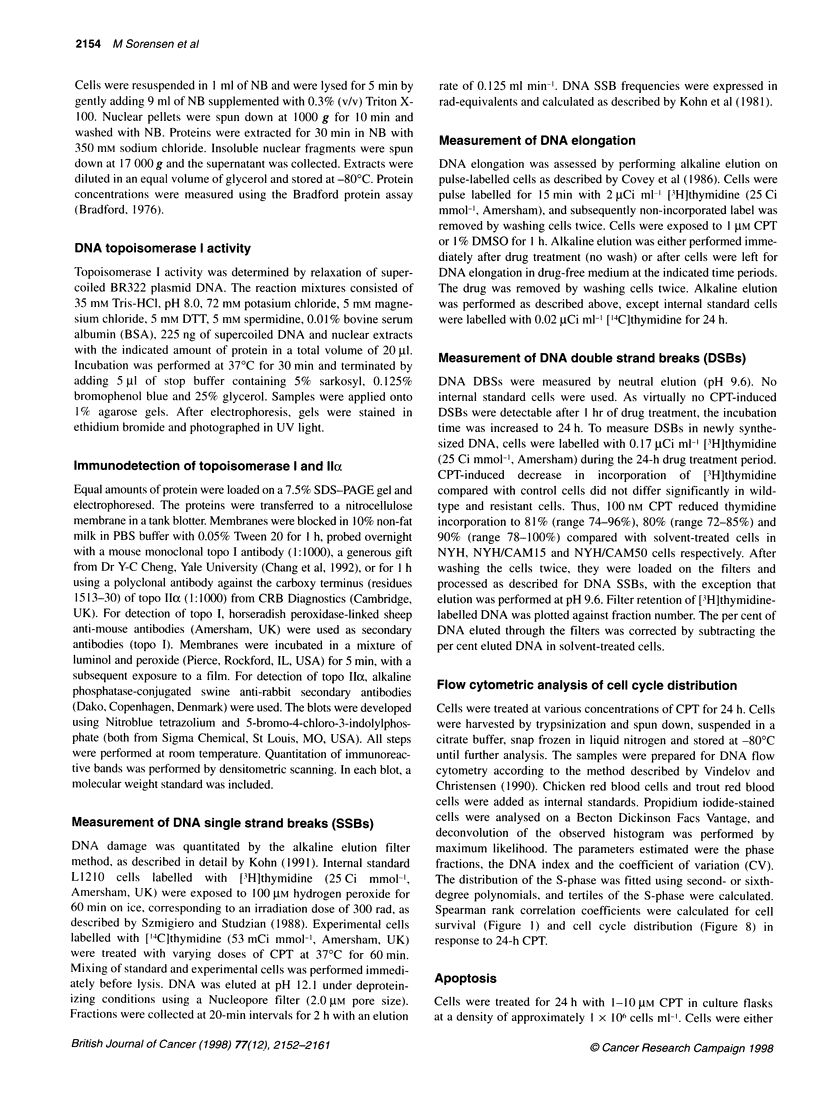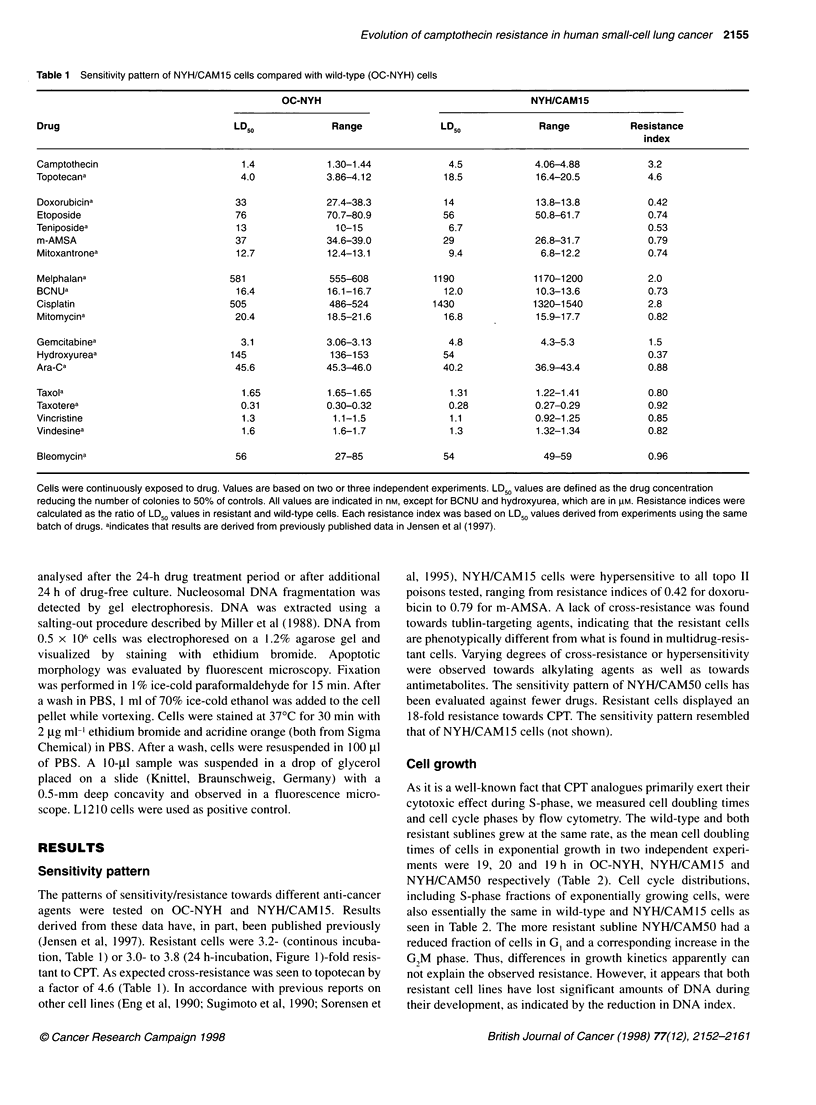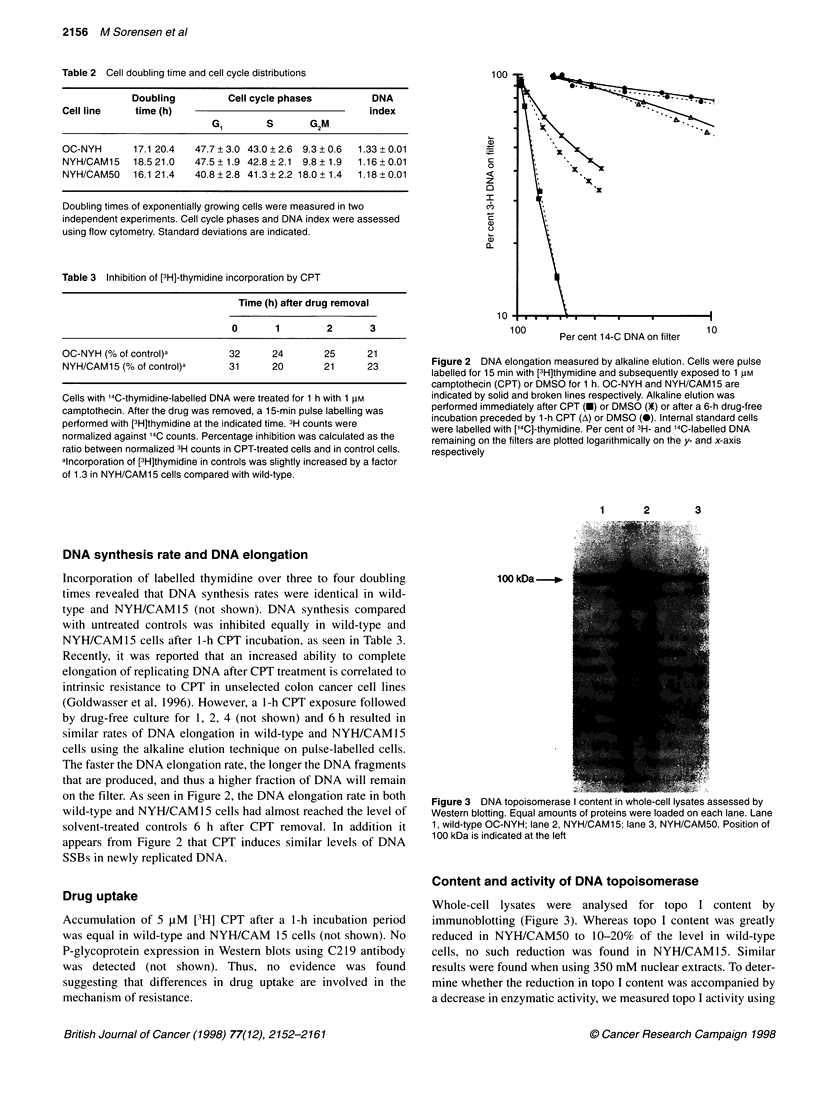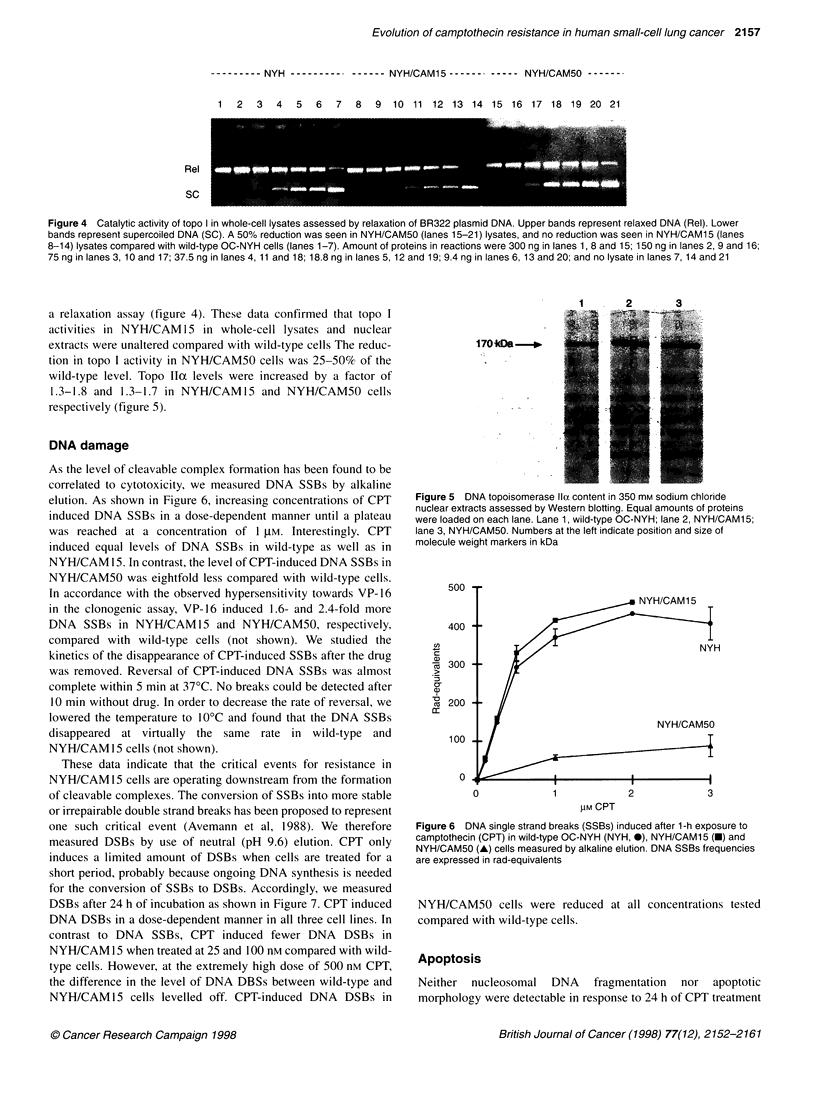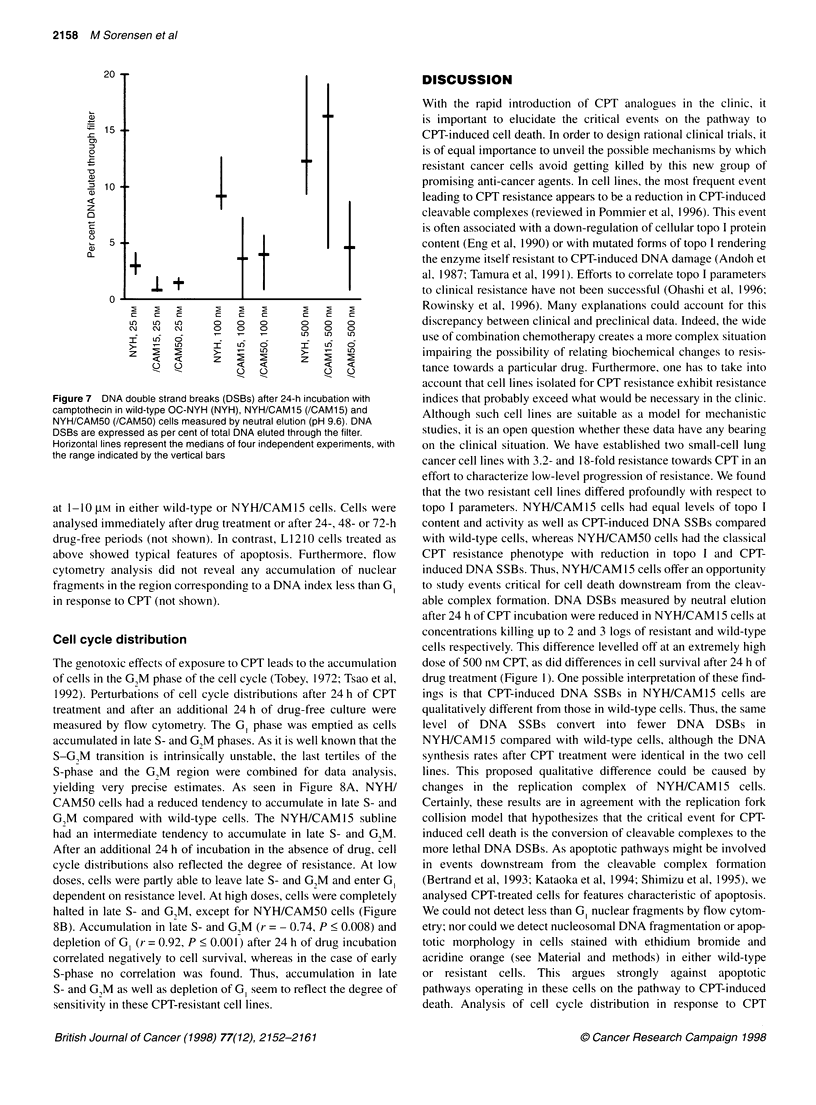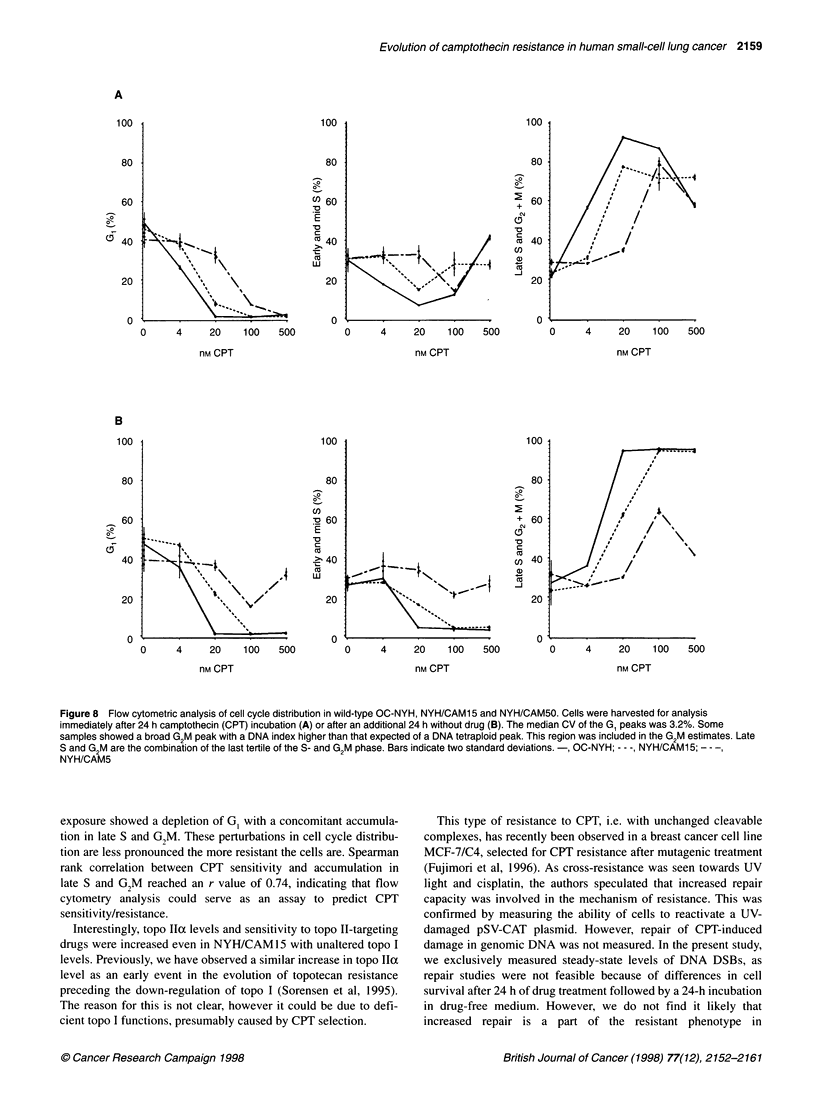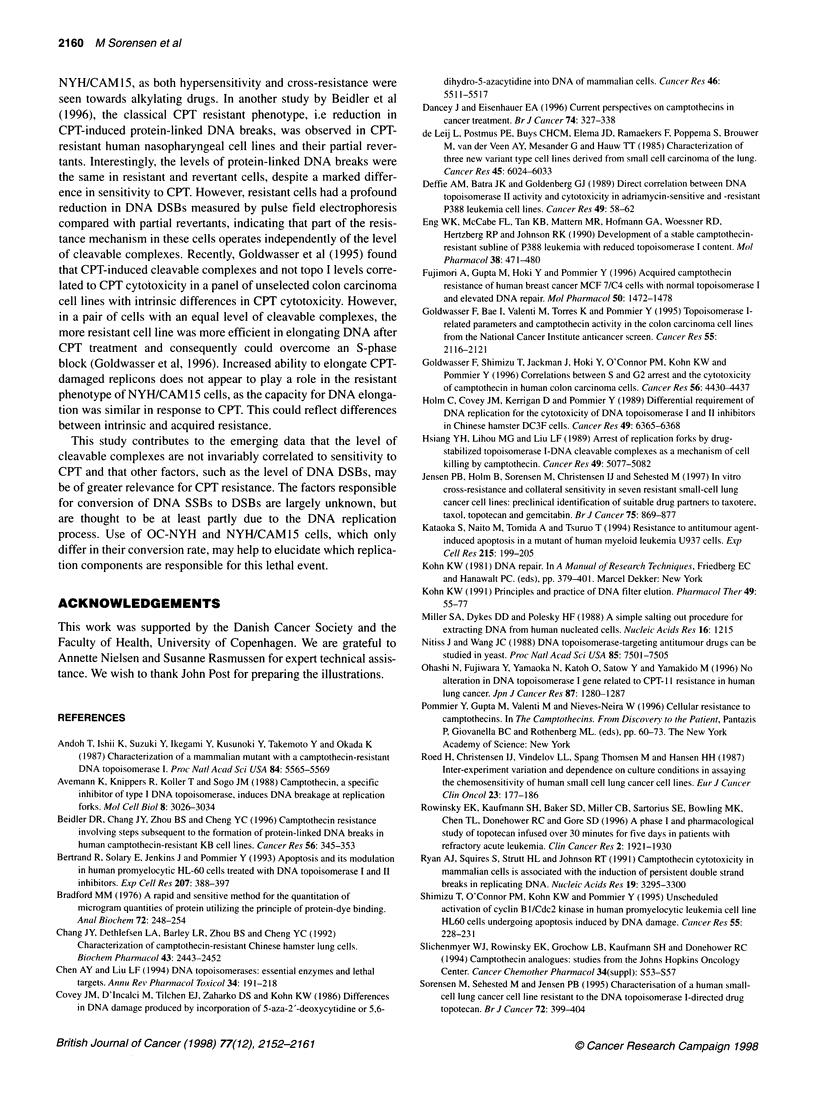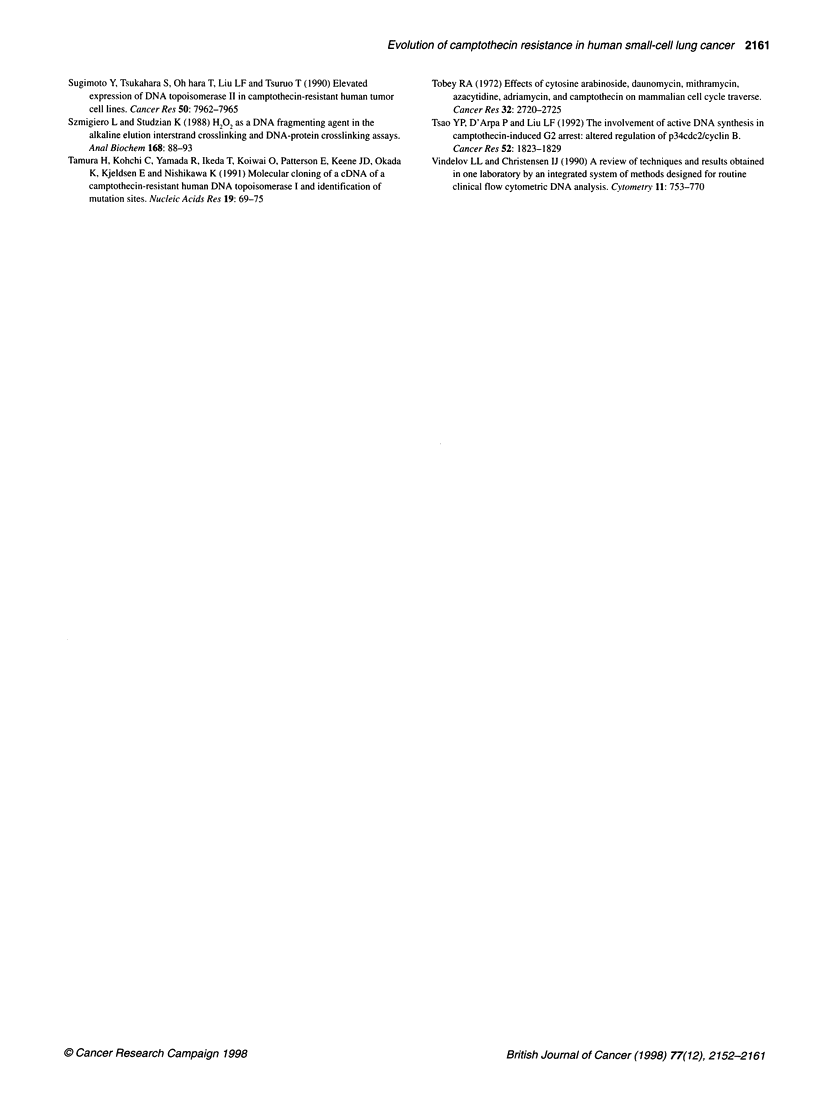# Low-level resistance to camptothecin in a human small-cell lung cancer cell line without reduction in DNA topoisomerase I or drug-induced cleavable complex formation.

**DOI:** 10.1038/bjc.1998.360

**Published:** 1998-06

**Authors:** M. Sorensen, M. Sehested, I. J. Christensen, J. K. Larsen, P. B. Jensen

**Affiliations:** Laboratory of Experimental Medical Oncology, The Finsen Center, Rigshospitalet, Copenhagen, Denmark.

## Abstract

**Images:**


British Journal of Cancer (1998) 77(12), 2152–2161
© 1998 Cancer Research Campaign

### Low-level resistance to camptothecin in a human small-cell lung cancer cell line without reduction in DNA topoisomerase I or drug-induced cleavable complex formation

**M Sorensen[1,2], M Sehested[2], IJ Christensen[3], JK Larsen[3] and PB Jensen[1]**

[1]Laboratory of Experimental Medical Oncology, The Finsen Center, Rigshospitalet, 9 Blegdamsvej, DK-2100 Copenhagen, Denmark; [2]Department of Pathology, The Laboratory Center, Rigshospitalet, DK-2100 Copenhagen, Denmark; [3]The Finsen Laboratory, Rigshospitalet, DK-2100 Copenhagen, Denmark

**Summary** To study the evolution of camptothecin (CPT) resistance, we have established two small-cell lung cancer cell lines with low (3.2-fold, NYH/CAM15) and high (18-fold, NYH/CAM50) resistance to CPT by stepwise drug exposure. NYH/CAM50 cells had reduced topoisomerase I (topo I) content and activity, and consequently CPT-induced DNA single strand breaks (SSBs) were reduced, as measured by alkaline elution. In contrast, NYH/CAM15 cells had identical topo I content and activity as compared with wild-type (wt) cells. CPT-mediated SSBs and the rate of their reversal after drug removal were also equal in wt and NYH/CAM15 cells, as were doubling time, the fraction of cells in S-phase and DNA synthesis rate in response to CPT. As the conversion of DNA SSBs to DNA double strand breaks (DSBs) is thought to represent a critical event leading to cell death, we measured DNA DSBs by neutral elution. In contrast to DNA SSBs, CPT induced fewer DNA DSBs in NYH/CAM15 than in wt cells. DNA flow cytometry showed that, in CPT-treated cells, the $G_1$ phase was emptied as cells accumulated in late S- and $G_2M$ phase. A Spearman rank correlation showed that depletion of $G_1$ and accumulation in late S and $G_2M$ correlated to CPT sensitivity in these three cell lines. In conclusion, acquired resistance to CPT can occur without a reduction in either topo I enzyme or CPT-induced cleavable complex formation, while a decrease in the level of CPT-induced DNA DSBs may be of major importance in the early stages of CPT resistance.

**Keywords:** resistance to camptothecin; DNA topoisomerase I; cleavable complex; DNA double strand breaks; cell cycle phase; small-cell lung cancer

The camptothecins (CPTs), which poison DNA topoisomerase (topo) I, are rapidly entering clinical trials (Slichenmyer et al, 1994; Dancey and Eisenhauer, 1996). Topo I relieves the torsional strain that accumulates as DNA replication and transcription occur. CPT stabilizes topo I-linked DNA single strand breaks (SSBs) also designated the cleavable complex (reviewed in Chen and Liu, 1994). The formation of these DNA SSBs is a prerequisite for CPT-induced cytotoxicity. However, DNA SSBs are not by themselves sufficient to kill cells. Several studies indicate that other factors downstream from DNA SSBs are crucial to the induction of cell death, including DNA synthesis (Holm et al, 1989) and repair processes (Nitiss and Wang, 1988). It has been hypothesized that the collision of cleavable complexes with advancing replication forks results in the conversion of transient DNA SSBs into the more lethal double strand breaks (DSBs) (Ryan et al, 1991) accompanied by replication fork arrest (Avemann et al, 1988). These DNA DSBs are thought to represent a critical event in CPT cytotoxicity, as their repair is hampered by the lack of template.

*Received 5 August 1997*
*Revised 10 December 1997*
*Accepted 17 January 1998*

*Correspondence to:* M Sorensen, Laboratory of Experimental Medical Oncology, The Finsen Center, 5074, Rigshospitalet, 9 Blegdamsvej, DK-2100 Copenhagen, Denmark

Several cell lines with acquired resistance to CPT have been characterized. To date, acquired resistance to CPT analogues has been associated with reduced formation of drug-induced DNA SSBs (reviewed in Pommier et al, 1996). This can be due to (1) decreased enzyme levels (Eng et al, 1990), (2) resistant forms of enzyme due to mutations (Andoh et al, 1987; Tamura et al, 1991) or (3) decreased accumulation of drug in rare cases (Chang et al, 1992). It is still an open question whether these observations translate to the clinical situation. Thus, studies on patients with adult leukaemia failed to show any relationship between topo I levels and clinical response to topotecan (Rowinsky al, 1996). Further, in 56 cases of lung cancer including eight patients exposed to CPT analogues, no topo I mutations were detected and the mean level of topo I mRNA was the same in those treated with or without CPT (Ohashi et al, 1996). It therefore remains to be documented that changes in topo I parameters or topo I mutations confer clinical resistance to CPT. There are several possible explanations for these disappointing results, one being the limited clinical tumour material available, severely hampering such studies. However, one must also consider that CPT-resistant cell lines characterized to date exhibit resistance indices of 7–300, which are probably well above what will be reached in the clinical situation. To investigate the progression of CPT resistance, we have established two CPT-resistant SCLC cell lines by stepwise exposure to drug: a low-level resistant line, NYH/CAM15, exhibiting 3.2-fold resistance and NYH/CAM50 with 18-fold resistance to CPT.

### METHODS AND MATERIALS

### Cell lines

The human SCLC cell line OC-NYH (also designated GLC-2) (de Leij et al, 1985) grows as a loosely attached monolayer in RPMI 1640 medium supplemented with 10% fetal calf serum plus penicillin, streptomycin and L-glutamine at 37°C in a humidified atmosphere with 7.5% carbon dioxide. The CPT-resistant subline NYH/CAM15 was established by exposing OC-NYH cells to increasing concentrations of drug during 9 months. Resistance was maintained by culturing cells in the presence of 15 nM CPT in every third passage. NYH/CAM50 cells were established by exposing NYH/CAM15 cells to CPT for an additional 8 months and maintained at 50 nM CPT in every third passage. Cells were grown without drug for at least two passages before experiments were performed. In order to avoid genetic drift, the cell lines used in experiments were re-established from frozen stocks at regular intervals (maximum of ten passages). Cell lines regularly tested negative for mycoplasma contamination.

### Clonogenic assay

Drug sensitivity was assessed by colony formation in soft agar with a feeder layer containing sheep red blood cells as previously described (Roed et al, 1987). Sensitivity patterns depicted in Table 1 were obtained in two or three independent experiments. Solvent concentrations never exceeded 1% and had no influence on the plating efficiency. Single-cell suspensions ($2 \times 10^4$ cells $ml^{-1}$) in RPMI 1640 supplemented with 10% fetal calf serum were plated in triplicate in the presence of drugs (continuous incubation). Fresh drug solutions were used in the clonogenic assay. CPT (Sigma), taxol (Bristol-Myers Squibb) and taxotere (Rhone-Poulenc Rohrer) were dissolved in dimethyl sulphoxide (DMSO). Topotecan (SmithKline Beecham), vincristine, gemcitabine (both from Lilly), doxorubicin, cytosine arabinoside (both from PharmaciaUpjohn), bleomycin (Lundbeck), hydroxyurea (Bristol-Myers Squibb) and mitomycin C (Kyowa) were dissolved in sterile water. Vindesine (Lilly) was dissolved in isotonic sodium chloride. BCNU (Bristol-Myers Squibb) was dissolved in 10% (v/v) ethanol. Melphalan (Welcome) was dissolved in hydrochloric acid with ethanol and further diluted in propyleneglycol phosphate buffer. m-AMSA (Parke-Davis) delivered in *N,N*-dimethylacetamid solution was further diluted in acid lactose. Etoposide, teniposide, cisplatin (all from Bristol-Myers Squibb) and mitoxantrone (Lederle) were in solution for infusion. After 14–21 days, the colonies were counted. Approximately 3000–4000 colonies were obtained in the control dishes. The level of resistance was calculated as the ratio between $LD_{50}$ values (the dose reducing the number of colonies to 50% of controls) for resistant and wild-type cells. The $LD_{50}$ values used for calculating resistance indices were derived from experiments with the use of the same batch of drugs. Survival curves in Figure 1 were obtained as described above, except cells were exposed to CPT for 24 h in RPMI 1640 supplemented with 10% fetal calf serum. After drug exposure, cells were washed twice in phosphate-buffered saline (PBS) and plated as above. Approximately 10 000 colonies were obtained in the control dishes.

**Figure 1** Survival curves of wild-type (NYH), NYH/CAM15 and NYH/CAM50 cells assessed by a clonogenic assay. After a 24-h treatment with camptothecin (CPT), cells were washed twice and plated in soft agar. Bars indicate s.e.m. of triplicate cultures

### DNA synthesis

Bulk DNA was labelled by culturing exponentially growing cells in the presence of 3 nCi $ml^{-1}$ [$^{14}C$]thymidine (53 mCi $mmol^{-1}$, Amersham, UK). After 3 days, the thymidine label was removed by washing cells twice in PBS with a subsequent chase in label-free medium for 24 h. Then, cells were harvested and were treated with 1 µM CPT or 1% DMSO as control for 60 min at 37°C in a 2-ml suspension of $1 \times 10^6$ cells $ml^{-1}$. The drug was removed by washing cells twice and cells were resuspended in 2 ml of drug-free medium. At the indicated time, cells were pulse labelled for 15 min with 1 µCi $ml^{-1}$ [$^3H$]thymidine (25 Ci $mmol^{-1}$, Amersham, UK). Precipitation was performed by adding a mixture of 2 ml of ice-cold PBS and 2 ml of ice-cold 10% trichloroacetic acid followed by centrifugation at 1200 r.p.m. for 5 min. After repeating the precipitation step, precipitates were dissolved in 800 µl of 0.3 N sodium hydroxide at 70°C for 30 min. $^3H$ counts were normalized against $^{14}C$ counts, and the percentage inhibition was calculated as the ratio between normalized $^3H$ counts in CPT-treated cells and that in control cells.

### Whole-cell lysates

Exponentially growing cells were harvested and washed once in phosphate-buffered saline. The cell pellet was lysed in buffer with 50 mM Hepes, pH 7.0, 250 mM sodium chloride, 5 mM EDTA and 0.1% (v/v) NP-40. Buffer volume was approximately ten times the volume of the pellet. After 30-min incubation on ice, insoluble cell fragments were spun down at 20 000 *g* for 20 min at 4°C. The supernatant was recovered and protein concentration was measured using the method of Bradford (Bradford, 1976)

### Crude nuclear extracts

Extracts were prepared as previously described with minor modifications (Deffie et al, 1989). All steps were performed at 4°C. Exponentially growing cells were harvested and washed twice in nucleus buffer (NB), (2 mM potassium dihydrogen phosphate, 5 mM magnesium chloride, 150 mM sodium chloride, 1 mM EGTA and 0.2 mM DTT, 1 mM phenylmethylsulphonyl fluoride, pH 6.5).

© Cancer Research Campaign 1998

Cells were resuspended in 1 ml of NB and were lysed for 5 min by gently adding 9 ml of NB supplemented with 0.3% (v/v) Triton X-100. Nuclear pellets were spun down at 1000 **g** for 10 min and washed with NB. Proteins were extracted for 30 min in NB with 350 mM sodium chloride. Insoluble nuclear fragments were spun down at 17 000 **g** and the supernatant was collected. Extracts were diluted in an equal volume of glycerol and stored at –80°C. Protein concentrations were measured using the Bradford protein assay (Bradford, 1976).

### DNA topoisomerase I activity

Topoisomerase I activity was determined by relaxation of supercoiled BR322 plasmid DNA. The reaction mixtures consisted of 35 mM Tris-HCl, pH 8.0, 72 mM potasium chloride, 5 mM magnesium chloride, 5 mM DTT, 5 mM spermidine, 0.01% bovine serum albumin (BSA), 225 ng of supercoiled DNA and nuclear extracts with the indicated amount of protein in a total volume of 20 µl. Incubation was performed at 37°C for 30 min and terminated by adding 5 µl of stop buffer containing 5% sarkosyl, 0.125% bromophenol blue and 25% glycerol. Samples were applied onto 1% agarose gels. After electrophoresis, gels were stained in ethidium bromide and photographed in UV light.

### Immunodetection of topoisomerase I and IIα

Equal amounts of protein were loaded on a 7.5% SDS–PAGE gel and electrophoresed. The proteins were transferred to a nitrocellulose membrane in a tank blotter. Membranes were blocked in 10% non-fat milk in PBS buffer with 0.05% Tween 20 for 1 h, probed overnight with a mouse monoclonal topo I antibody (1:1000), a generous gift from Dr Y-C Cheng, Yale University (Chang et al, 1992), or for 1 h using a polyclonal antibody against the carboxy terminus (residues 1513–30) of topo IIα (1:1000) from CRB Diagnostics (Cambridge, UK). For detection of topo I, horseradish peroxidase-linked sheep anti-mouse antibodies (Amersham, UK) were used as secondary antibodies (topo I). Membranes were incubated in a mixture of luminol and peroxide (Pierce, Rockford, IL, USA) for 5 min, with a subsequent exposure to a film. For detection of topo IIα, alkaline phosphatase-conjugated swine anti-rabbit secondary antibodies (Dako, Copenhagen, Denmark) were used. The blots were developed using Nitroblue tetrazolium and 5-bromo-4-chloro-3-indolylphosphate (both from Sigma Chemical, St Louis, MO, USA). All steps were performed at room temperature. Quantitation of immunoreactive bands was performed by densitometric scanning. In each blot, a molecular weight standard was included.

### Measurement of DNA single strand breaks (SSBs)

DNA damage was quantitated by the alkaline elution filter method, as described in detail by Kohn (1991). Internal standard L1210 cells labelled with [$^3$H]thymidine (25 Ci mmol$^{-1}$, Amersham, UK) were exposed to 100 µM hydrogen peroxide for 60 min on ice, corresponding to an irradiation dose of 300 rad, as described by Szmigiero and Studzian (1988). Experimental cells labelled with [$^{14}$C]thymidine (53 mCi mmol$^{-1}$, Amersham, UK) were treated with varying doses of CPT at 37°C for 60 min. Mixing of standard and experimental cells was performed immediately before lysis. DNA was eluted at pH 12.1 under deproteinizing conditions using a Nucleopore filter (2.0 µM pore size). Fractions were collected at 20-min intervals for 2 h with an elution rate of 0.125 ml min$^{-1}$. DNA SSB frequencies were expressed in rad-equivalents and calculated as described by Kohn et al (1981).

### Measurement of DNA elongation

DNA elongation was assessed by performing alkaline elution on pulse-labelled cells as described by Covey et al (1986). Cells were pulse labelled for 15 min with 2 µCi ml$^{-1}$ [$^3$H]thymidine (25 Ci mmol$^{-1}$, Amersham), and subsequently non-incorporated label was removed by washing cells twice. Cells were exposed to 1 µM CPT or 1% DMSO for 1 h. Alkaline elution was either performed immediately after drug treatment (no wash) or after cells were left for DNA elongation in drug-free medium at the indicated time periods. The drug was removed by washing cells twice. Alkaline elution was performed as described above, except internal standard cells were labelled with 0.02 µCi ml$^{-1}$ [$^{14}$C]thymidine for 24 h.

### Measurement of DNA double strand breaks (DSBs)

DNA DBSs were measured by neutral elution (pH 9.6). No internal standard cells were used. As virtually no CPT-induced DSBs were detectable after 1 hr of drug treatment, the incubation time was increased to 24 h. To measure DSBs in newly synthesized DNA, cells were labelled with 0.17 µCi ml$^{-1}$ [$^3$H]thymidine (25 Ci mmol$^{-1}$, Amersham) during the 24-h drug treatment period. CPT-induced decrease in incorporation of [$^3$H]thymidine compared with control cells did not differ significantly in wild-type and resistant cells. Thus, 100 nM CPT reduced thymidine incorporation to 81% (range 74–96%), 80% (range 72–85%) and 90% (range 78–100%) compared with solvent-treated cells in NYH, NYH/CAM15 and NYH/CAM50 cells respectively. After washing the cells twice, they were loaded on the filters and processed as described for DNA SSBs, with the exception that elution was performed at pH 9.6. Filter retention of [$^3$H]thymidine-labelled DNA was plotted against fraction number. The per cent of DNA eluted through the filters was corrected by subtracting the per cent eluted DNA in solvent-treated cells.

### Flow cytometric analysis of cell cycle distribution

Cells were treated at various concentrations of CPT for 24 h. Cells were harvested by trypsinization and spun down, suspended in a citrate buffer, snap frozen in liquid nitrogen and stored at –80°C until further analysis. The samples were prepared for DNA flow cytometry according to the method described by Vindelov and Christensen (1990). Chicken red blood cells and trout red blood cells were added as internal standards. Propidium iodide-stained cells were analysed on a Becton Dickinson Facs Vantage, and deconvolution of the observed histogram was performed by maximum likelihood. The parameters estimated were the phase fractions, the DNA index and the coefficient of variation (CV). The distribution of the S-phase was fitted using second- or sixth-degree polynomials, and tertiles of the S-phase were calculated. Spearman rank correlation coefficients were calculated for cell survival (Figure 1) and cell cycle distribution (Figure 8) in response to 24-h CPT.

### Apoptosis

Cells were treated for 24 h with 1–10 µM CPT in culture flasks at a density of approximately 1 × 10$^6$ cells ml$^{-1}$. Cells were either

© Cancer Research Campaign 1998

**Table 1** Sensitivity pattern of NYH/CAM15 cells compared with wild-type (OC-NYH) cells

| Drug | OC-NYH | | NYH/CAM15 | | |
|---|---|---|---|---|---|
| | $LD_{50}$ | Range | $LD_{50}$ | Range | Resistance index |
| Camptothecin | 1.4 | 1.30–1.44 | 4.5 | 4.06–4.88 | 3.2 |
| Topotecan[a] | 4.0 | 3.86–4.12 | 18.5 | 16.4–20.5 | 4.6 |
| Doxorubicin[a] | 33 | 27.4–38.3 | 14 | 13.8–13.8 | 0.42 |
| Etoposide | 76 | 70.7–80.9 | 56 | 50.8–61.7 | 0.74 |
| Teniposide[a] | 13 | 10–15 | 6.7 | | 0.53 |
| m-AMSA | 37 | 34.6–39.0 | 29 | 26.8–31.7 | 0.79 |
| Mitoxantrone[a] | 12.7 | 12.4–13.1 | 9.4 | 6.8–12.2 | 0.74 |
| Melphalan[a] | 581 | 555–608 | 1190 | 1170–1200 | 2.0 |
| BCNU[a] | 16.4 | 16.1–16.7 | 12.0 | 10.3–13.6 | 0.73 |
| Cisplatin | 505 | 486–524 | 1430 | 1320–1540 | 2.8 |
| Mitomycin[a] | 20.4 | 18.5–21.6 | 16.8 | 15.9–17.7 | 0.82 |
| Gemcitabine[a] | 3.1 | 3.06–3.13 | 4.8 | 4.3–5.3 | 1.5 |
| Hydroxyurea[a] | 145 | 136–153 | 54 | | 0.37 |
| Ara-C[a] | 45.6 | 45.3–46.0 | 40.2 | 36.9–43.4 | 0.88 |
| Taxol[a] | 1.65 | 1.65–1.65 | 1.31 | 1.22–1.41 | 0.80 |
| Taxotere[a] | 0.31 | 0.30–0.32 | 0.28 | 0.27–0.29 | 0.92 |
| Vincristine | 1.3 | 1.1–1.5 | 1.1 | 0.92–1.25 | 0.85 |
| Vindesine[a] | 1.6 | 1.6–1.7 | 1.3 | 1.32–1.34 | 0.82 |
| Bleomycin[a] | 56 | 27–85 | 54 | 49–59 | 0.96 |

Cells were continuously exposed to drug. Values are based on two or three independent experiments. $LD_{50}$ values are defined as the drug concentration reducing the number of colonies to 50% of controls. All values are indicated in nM, except for BCNU and hydroxyurea, which are in μM. Resistance indices were calculated as the ratio of $LD_{50}$ values in resistant and wild-type cells. Each resistance index was based on $LD_{50}$ values derived from experiments using the same batch of drugs. [a]indicates that results are derived from previously published data in Jensen et al (1997).

analysed after the 24-h drug treatment period or after additional 24 h of drug-free culture. Nucleosomal DNA fragmentation was detected by gel electrophoresis. DNA was extracted using a salting-out procedure described by Miller et al (1988). DNA from 0.5 × $10^6$ cells was electrophoresed on a 1.2% agarose gel and visualized by staining with ethidium bromide. Apoptotic morphology was evaluated by fluorescent microscopy. Fixation was performed in 1% ice-cold paraformaldehyde for 15 min. After a wash in PBS, 1 ml of 70% ice-cold ethanol was added to the cell pellet while vortexing. Cells were stained at 37°C for 30 min with 2 μg $ml^{-1}$ ethidium bromide and acridine orange (both from Sigma Chemical) in PBS. After a wash, cells were resuspended in 100 μl of PBS. A 10-μl sample was suspended in a drop of glycerol placed on a slide (Knittel, Braunschweig, Germany) with a 0.5-mm deep concavity and observed in a fluorescence microscope. L1210 cells were used as positive control.

### RESULTS

### Sensitivity pattern

The patterns of sensitivity/resistance towards different anti-cancer agents were tested on OC-NYH and NYH/CAM15. Results derived from these data have, in part, been published previously (Jensen et al, 1997). Resistant cells were 3.2- (continous incubation, Table 1) or 3.0- to 3.8 (24 h-incubation, Figure 1)-fold resistant to CPT. As expected cross-resistance was seen to topotecan by a factor of 4.6 (Table 1). In accordance with previous reports on other cell lines (Eng et al, 1990; Sugimoto et al, 1990; Sorensen et al, 1995), NYH/CAM15 cells were hypersensitive to all topo II poisons tested, ranging from resistance indices of 0.42 for doxorubicin to 0.79 for m-AMSA. A lack of cross-resistance was found towards tublin-targeting agents, indicating that the resistant cells are phenotypically different from what is found in multidrug-resistant cells. Varying degrees of cross-resistance or hypersensitivity were observed towards alkylating agents as well as towards antimetabolites. The sensitivity pattern of NYH/CAM50 cells has been evaluated against fewer drugs. Resistant cells displayed an 18-fold resistance towards CPT. The sensitivity pattern resembled that of NYH/CAM15 cells (not shown).

### Cell growth

As it is a well-known fact that CPT analogues primarily exert their cytotoxic effect during S-phase, we measured cell doubling times and cell cycle phases by flow cytometry. The wild-type and both resistant sublines grew at the same rate, as the mean cell doubling times of cells in exponential growth in two independent experiments were 19, 20 and 19 h in OC-NYH, NYH/CAM15 and NYH/CAM50 respectively (Table 2). Cell cycle distributions, including S-phase fractions of exponentially growing cells, were also essentially the same in wild-type and NYH/CAM15 cells as seen in Table 2. The more resistant subline NYH/CAM50 had a reduced fraction of cells in $G_1$ and a corresponding increase in the $G_2M$ phase. Thus, differences in growth kinetics apparently can not explain the observed resistance. However, it appears that both resistant cell lines have lost significant amounts of DNA during their development, as indicated by the reduction in DNA index.

**Table 2** Cell doubling time and cell cycle distributions

| Cell line | Doubling time (h) | Cell cycle phases | | | DNA index |
|---|---|---|---|---|---|
| | | $G_1$ | S | $G_2M$ | |
| OC-NYH | 17.1 20.4 | 47.7 ± 3.0 | 43.0 ± 2.6 | 9.3 ± 0.6 | 1.33 ± 0.01 |
| NYH/CAM15 | 18.5 21.0 | 47.5 ± 1.9 | 42.8 ± 2.1 | 9.8 ± 1.9 | 1.16 ± 0.01 |
| NYH/CAM50 | 16.1 21.4 | 40.8 ± 2.8 | 41.3 ± 2.2 | 18.0 ± 1.4 | 1.18 ± 0.01 |

Doubling times of exponentially growing cells were measured in two independent experiments. Cell cycle phases and DNA index were assessed using flow cytometry. Standard deviations are indicated.

**Table 3** Inhibition of [$^3$H]-thymidine incorporation by CPT

| | Time (h) after drug removal | | | |
|---|---|---|---|---|
| | 0 | 1 | 2 | 3 |
| OC-NYH (% of control)[a] | 32 | 24 | 25 | 21 |
| NYH/CAM15 (% of control)[a] | 31 | 20 | 21 | 23 |

Cells with $^{14}$C-thymidine-labelled DNA were treated for 1 h with 1 μM camptothecin. After the drug was removed, a 15-min pulse labelling was performed with [$^3$H]thymidine at the indicated time. $^3$H counts were normalized against $^{14}$C counts. Percentage inhibition was calculated as the ratio between normalized $^3$H counts in CPT-treated cells and in control cells. [a]Incorporation of [$^3$H]thymidine in controls was slightly increased by a factor of 1.3 in NYH/CAM15 cells compared with wild-type.

**Figure 2** DNA elongation measured by alkaline elution. Cells were pulse labelled for 15 min with [$^3$H]thymidine and subsequently exposed to 1 μM camptothecin (CPT) or DMSO for 1 h. OC-NYH and NYH/CAM15 are indicated by solid and broken lines respectively. Alkaline elution was performed immediately after CPT (■) or DMSO (✱) or after a 6-h drug-free incubation preceded by 1-h CPT (Δ) or DMSO (●). Internal standard cells were labelled with [$^{14}$C]-thymidine. Per cent of $^3$H- and $^{14}$C-labelled DNA remaining on the filters are plotted logarithmically on the *y*- and *x*-axis respectively

**Figure 3** DNA topoisomerase I content in whole-cell lysates assessed by Western blotting. Equal amounts of proteins were loaded on each lane. Lane 1, wild-type OC-NYH; lane 2, NYH/CAM15; lane 3, NYH/CAM50. Position of 100 kDa is indicated at the left

### DNA synthesis rate and DNA elongation

Incorporation of labelled thymidine over three to four doubling times revealed that DNA synthesis rates were identical in wild-type and NYH/CAM15 (not shown). DNA synthesis compared with untreated controls was inhibited equally in wild-type and NYH/CAM15 cells after 1-h CPT incubation, as seen in Table 3. Recently, it was reported that an increased ability to complete elongation of replicating DNA after CPT treatment is correlated to intrinsic resistance to CPT in unselected colon cancer cell lines (Goldwasser et al, 1996). However, a 1-h CPT exposure followed by drug-free culture for 1, 2, 4 (not shown) and 6 h resulted in similar rates of DNA elongation in wild-type and NYH/CAM15 cells using the alkaline elution technique on pulse-labelled cells. The faster the DNA elongation rate, the longer the DNA fragments that are produced, and thus a higher fraction of DNA will remain on the filter. As seen in Figure 2, the DNA elongation rate in both wild-type and NYH/CAM15 cells had almost reached the level of solvent-treated controls 6 h after CPT removal. In addition it appears from Figure 2 that CPT induces similar levels of DNA SSBs in newly replicated DNA.

### Drug uptake

Accumulation of 5 μM [$^3$H] CPT after a 1-h incubation period was equal in wild-type and NYH/CAM 15 cells (not shown). No P-glycoprotein expression in Western blots using C219 antibody was detected (not shown). Thus, no evidence was found suggesting that differences in drug uptake are involved in the mechanism of resistance.

### Content and activity of DNA topoisomerase

Whole-cell lysates were analysed for topo I content by immunoblotting (Figure 3). Whereas topo I content was greatly reduced in NYH/CAM50 to 10–20% of the level in wild-type cells, no such reduction was found in NYH/CAM15. Similar results were found when using 350 mM nuclear extracts. To determine whether the reduction in topo I content was accompanied by a decrease in enzymatic activity, we measured topo I activity using

 © Cancer Research Campaign 1998

**Figure 4** Catalytic activity of topo I in whole-cell lysates assessed by relaxation of BR322 plasmid DNA. Upper bands represent relaxed DNA (Rel). Lower bands represent supercoiled DNA (SC). A 50% reduction was seen in NYH/CAM50 (lanes 15–21) lysates, and no reduction was seen in NYH/CAM15 (lanes 8–14) lysates compared with wild-type OC-NYH cells (lanes 1–7). Amount of proteins in reactions were 300 ng in lanes 1, 8 and 15; 150 ng in lanes 2, 9 and 16; 75 ng in lanes 3, 10 and 17; 37.5 ng in lanes 4, 11 and 18; 18.8 ng in lanes 5, 12 and 19; 9.4 ng in lanes 6, 13 and 20; and no lysate in lanes 7, 14 and 21

a relaxation assay (figure 4). These data confirmed that topo I activities in NYH/CAM15 in whole-cell lysates and nuclear extracts were unaltered compared with wild-type cells The reduction in topo I activity in NYH/CAM50 cells was 25–50% of the wild-type level. Topo IIα levels were increased by a factor of 1.3–1.8 and 1.3–1.7 in NYH/CAM15 and NYH/CAM50 cells respectively (figure 5).

### DNA damage

As the level of cleavable complex formation has been found to be correlated to cytotoxicity, we measured DNA SSBs by alkaline elution. As shown in Figure 6, increasing concentrations of CPT induced DNA SSBs in a dose-dependent manner until a plateau was reached at a concentration of 1 μM. Interestingly, CPT induced equal levels of DNA SSBs in wild-type as well as in NYH/CAM15. In contrast, the level of CPT-induced DNA SSBs in NYH/CAM50 was eightfold less compared with wild-type cells. In accordance with the observed hypersensitivity towards VP-16 in the clonogenic assay, VP-16 induced 1.6- and 2.4-fold more DNA SSBs in NYH/CAM15 and NYH/CAM50, respectively, compared with wild-type cells (not shown). We studied the kinetics of the disappearance of CPT-induced SSBs after the drug was removed. Reversal of CPT-induced DNA SSBs was almost complete within 5 min at 37°C. No breaks could be detected after 10 min without drug. In order to decrease the rate of reversal, we lowered the temperature to 10°C and found that the DNA SSBs disappeared at virtually the same rate in wild-type and NYH/CAM15 cells (not shown).

These data indicate that the critical events for resistance in NYH/CAM15 cells are operating downstream from the formation of cleavable complexes. The conversion of SSBs into more stable or irrepairable double strand breaks has been proposed to represent one such critical event (Avemann et al, 1988). We therefore measured DSBs by use of neutral (pH 9.6) elution. CPT only induces a limited amount of DSBs when cells are treated for a short period, probably because ongoing DNA synthesis is needed for the conversion of SSBs to DSBs. Accordingly, we measured DSBs after 24 h of incubation as shown in Figure 7. CPT induced DNA DSBs in a dose-dependent manner in all three cell lines. In contrast to DNA SSBs, CPT induced fewer DNA DSBs in NYH/CAM15 when treated at 25 and 100 nM compared with wild-type cells. However, at the extremely high dose of 500 nM CPT, the difference in the level of DNA DBSs between wild-type and NYH/CAM15 cells levelled off. CPT-induced DNA DSBs in NYH/CAM50 cells were reduced at all concentrations tested compared with wild-type cells.

**Figure 5** DNA topoisomerase IIα content in 350 mM sodium chloride nuclear extracts assessed by Western blotting. Equal amounts of proteins were loaded on each lane. Lane 1, wild-type OC-NYH; lane 2, NYH/CAM15; lane 3, NYH/CAM50. Numbers at the left indicate position and size of molecule weight markers in kDa

**Figure 6** DNA single strand breaks (SSBs) induced after 1-h exposure to camptothecin (CPT) in wild-type OC-NYH (NYH, ●), NYH/CAM15 (■) and NYH/CAM50 (▲) cells measured by alkaline elution. DNA SSBs frequencies are expressed in rad-equivalents

### Apoptosis

Neither nucleosomal DNA fragmentation nor apoptotic morphology were detectable in response to 24 h of CPT treatment

© Cancer Research Campaign 1998

**Figure 7** DNA double strand breaks (DSBs) after 24-h incubation with camptothecin in wild-type OC-NYH (NYH), NYH/CAM15 (/CAM15) and NYH/CAM50 (/CAM50) cells measured by neutral elution (pH 9.6). DNA DSBs are expressed as per cent of total DNA eluted through the filter. Horizontal lines represent the medians of four independent experiments, with the range indicated by the vertical bars at 1–10 μM in either wild-type or NYH/CAM15 cells. Cells were analysed immediately after drug treatment or after 24-, 48- or 72-h drug-free periods (not shown). In contrast, L1210 cells treated as above showed typical features of apoptosis. Furthermore, flow cytometry analysis did not reveal any accumulation of nuclear fragments in the region corresponding to a DNA index less than $G_1$ in response to CPT (not shown).

### Cell cycle distribution

The genotoxic effects of exposure to CPT leads to the accumulation of cells in the $G_2M$ phase of the cell cycle (Tobey, 1972; Tsao et al, 1992). Perturbations of cell cycle distributions after 24 h of CPT treatment and after an additional 24 h of drug-free culture were measured by flow cytometry. The $G_1$ phase was emptied as cells accumulated in late S- and $G_2M$ phases. As it is well known that the S–$G_2M$ transition is intrinsically unstable, the last tertiles of the S-phase and the $G_2M$ region were combined for data analysis, yielding very precise estimates. As seen in Figure 8A, NYH/CAM50 cells had a reduced tendency to accumulate in late S- and $G_2M$ compared with wild-type cells. The NYH/CAM15 subline had an intermediate tendency to accumulate in late S- and $G_2M$. After an additional 24 h of incubation in the absence of drug, cell cycle distributions also reflected the degree of resistance. At low doses, cells were partly able to leave late S- and $G_2M$ and enter $G_1$ dependent on resistance level. At high doses, cells were completely halted in late S- and $G_2M$, except for NYH/CAM50 cells (Figure 8B). Accumulation in late S- and $G_2M$ ($r = -0.74$, $P \leq 0.008$) and depletion of $G_1$ ($r = 0.92$, $P \leq 0.001$) after 24 h of drug incubation correlated negatively to cell survival, whereas in the case of early S-phase no correlation was found. Thus, accumulation in late S- and $G_2M$ as well as depletion of $G_1$ seem to reflect the degree of sensitivity in these CPT-resistant cell lines.

### DISCUSSION

With the rapid introduction of CPT analogues in the clinic, it is important to elucidate the critical events on the pathway to CPT-induced cell death. In order to design rational clinical trials, it is of equal importance to unveil the possible mechanisms by which resistant cancer cells avoid getting killed by this new group of promising anti-cancer agents. In cell lines, the most frequent event leading to CPT resistance appears to be a reduction in CPT-induced cleavable complexes (reviewed in Pommier et al, 1996). This event is often associated with a down-regulation of cellular topo I protein content (Eng et al, 1990) or with mutated forms of topo I rendering the enzyme itself resistant to CPT-induced DNA damage (Andoh et al, 1987; Tamura et al, 1991). Efforts to correlate topo I parameters to clinical resistance have not been successful (Ohashi et al, 1996; Rowinsky et al, 1996). Many explanations could account for this discrepancy between clinical and preclinical data. Indeed, the wide use of combination chemotherapy creates a more complex situation impairing the possibility of relating biochemical changes to resistance towards a particular drug. Furthermore, one has to take into account that cell lines isolated for CPT resistance exhibit resistance indices that probably exceed what would be necessary in the clinic. Although such cell lines are suitable as a model for mechanistic studies, it is an open question whether these data have any bearing on the clinical situation. We have established two small-cell lung cancer cell lines with 3.2- and 18-fold resistance towards CPT in an effort to characterize low-level progression of resistance. We found that the two resistant cell lines differed profoundly with respect to topo I parameters. NYH/CAM15 cells had equal levels of topo I content and activity as well as CPT-induced DNA SSBs compared with wild-type cells, whereas NYH/CAM50 cells had the classical CPT resistance phenotype with reduction in topo I and CPT-induced DNA SSBs. Thus, NYH/CAM15 cells offer an opportunity to study events critical for cell death downstream from the cleavable complex formation. DNA DSBs measured by neutral elution after 24 h of CPT incubation were reduced in NYH/CAM15 cells at concentrations killing up to 2 and 3 logs of resistant and wild-type cells respectively. This difference levelled off at an extremely high dose of 500 nM CPT, as did differences in cell survival after 24 h of drug treatment (Figure 1). One possible interpretation of these findings is that CPT-induced DNA SSBs in NYH/CAM15 cells are qualitatively different from those in wild-type cells. Thus, the same level of DNA SSBs convert into fewer DNA DSBs in NYH/CAM15 compared with wild-type cells, although the DNA synthesis rates after CPT treatment were identical in the two cell lines. This proposed qualitative difference could be caused by changes in the replication complex of NYH/CAM15 cells. Certainly, these results are in agreement with the replication fork collision model that hypothesizes that the critical event for CPT-induced cell death is the conversion of cleavable complexes to the more lethal DNA DSBs. As apoptotic pathways might be involved in events downstream from the cleavable complex formation (Bertrand et al, 1993; Kataoka et al, 1994; Shimizu et al, 1995), we analysed CPT-treated cells for features characteristic of apoptosis. We could not detect less than $G_1$ nuclear fragments by flow cytometry; nor could we detect nucleosomal DNA fragmentation or apoptotic morphology in cells stained with ethidium bromide and acridine orange (see Material and methods) in either wild-type or resistant cells. This argues strongly against apoptotic pathways operating in these cells on the pathway to CPT-induced death. Analysis of cell cycle distribution in response to CPT

© Cancer Research Campaign 1998

**Figure 8** Flow cytometric analysis of cell cycle distribution in wild-type OC-NYH, NYH/CAM15 and NYH/CAM50. Cells were harvested for analysis immediately after 24 h camptothecin (CPT) incubation (**A**) or after an additional 24 h without drug (**B**). The median CV of the $G_1$ peaks was 3.2%. Some samples showed a broad $G_2M$ peak with a DNA index higher than that expected of a DNA tetraploid peak. This region was included in the $G_2M$ estimates. Late S and $G_2M$ are the combination of the last tertile of the S- and $G_2M$ phase. Bars indicate two standard deviations. —, OC-NYH; - - -, NYH/CAM15; – – –, NYH/CAM5

exposure showed a depletion of $G_1$ with a concomitant accumulation in late S and $G_2M$. These perturbations in cell cycle distribution are less pronounced the more resistant the cells are. Spearman rank correlation between CPT sensitivity and accumulation in late S and $G_2M$ reached an $r$ value of 0.74, indicating that flow cytometry analysis could serve as an assay to predict CPT sensitivity/resistance.

Interestingly, topo IIα levels and sensitivity to topo II-targeting drugs were increased even in NYH/CAM15 with unaltered topo I levels. Previously, we have observed a similar increase in topo IIα level as an early event in the evolution of topotecan resistance preceding the down-regulation of topo I (Sorensen et al, 1995). The reason for this is not clear, however it could be due to deficient topo I functions, presumably caused by CPT selection.

This type of resistance to CPT, i.e. with unchanged cleavable complexes, has recently been observed in a breast cancer cell line MCF-7/C4, selected for CPT resistance after mutagenic treatment (Fujimori et al, 1996). As cross-resistance was seen towards UV light and cisplatin, the authors speculated that increased repair capacity was involved in the mechanism of resistance. This was confirmed by measuring the ability of cells to reactivate a UV-damaged pSV-CAT plasmid. However, repair of CPT-induced damage in genomic DNA was not measured. In the present study, we exclusively measured steady-state levels of DNA DSBs, as repair studies were not feasible because of differences in cell survival after 24 h of drug treatment followed by a 24-h incubation in drug-free medium. However, we do not find it likely that increased repair is a part of the resistant phenotype in

NYH/CAM15, as both hypersensitivity and cross-resistance were seen towards alkylating drugs. In another study by Beidler et al (1996), the classical CPT resistant phenotype, i.e reduction in CPT-induced protein-linked DNA breaks, was observed in CPT-resistant human nasopharyngeal cell lines and their partial revertants. Interestingly, the levels of protein-linked DNA breaks were the same in resistant and revertant cells, despite a marked difference in sensitivity to CPT. However, resistant cells had a profound reduction in DNA DSBs measured by pulse field electrophoresis compared with partial revertants, indicating that part of the resistance mechanism in these cells operates independently of the level of cleavable complexes. Recently, Goldwasser et al (1995) found that CPT-induced cleavable complexes and not topo I levels correlated to CPT cytotoxicity in a panel of unselected colon carcinoma cell lines with intrinsic differences in CPT cytotoxicity. However, in a pair of cells with an equal level of cleavable complexes, the more resistant cell line was more efficient in elongating DNA after CPT treatment and consequently could overcome an S-phase block (Goldwasser et al, 1996). Increased ability to elongate CPT-damaged replicons does not appear to play a role in the resistant phenotype of NYH/CAM15 cells, as the capacity for DNA elongation was similar in response to CPT. This could reflect differences between intrinsic and acquired resistance.

This study contributes to the emerging data that the level of cleavable complexes are not invariably correlated to sensitivity to CPT and that other factors, such as the level of DNA DSBs, may be of greater relevance for CPT resistance. The factors responsible for conversion of DNA SSBs to DSBs are largely unknown, but are thought to be at least partly due to the DNA replication process. Use of OC-NYH and NYH/CAM15 cells, which only differ in their conversion rate, may help to elucidate which replication components are responsible for this lethal event.

### ACKNOWLEDGEMENTS

This work was supported by the Danish Cancer Society and the Faculty of Health, University of Copenhagen. We are grateful to Annette Nielsen and Susanne Rasmussen for expert technical assistance. We wish to thank John Post for preparing the illustrations.

### REFERENCES

Andoh T, Ishii K, Suzuki Y, Ikegami Y, Kusunoki Y, Takemoto Y and Okada K (1987) Characterization of a mammalian mutant with a camptothecin-resistant DNA topoisomerase I. *Proc Natl Acad Sci USA* **84**: 5565–5569

Avemann K, Knippers R, Koller T and Sogo JM (1988) Camptothecin, a specific inhibitor of type I DNA topoisomerase, induces DNA breakage at replication forks. *Mol Cell Biol* **8**: 3026–3034

Beidler DR, Chang JY, Zhou BS and Cheng YC (1996) Camptothecin resistance involving steps subsequent to the formation of protein-linked DNA breaks in human camptothecin-resistant KB cell lines. *Cancer Res* **56**: 345–353

Bertrand R, Solary E, Jenkins J and Pommier Y (1993) Apoptosis and its modulation in human promyelocytic HL-60 cells treated with DNA topoisomerase I and II inhibitors. *Exp Cell Res* **207**: 388–397

Bradford MM (1976) A rapid and sensitive method for the quantitation of microgram quantities of protein utilizing the principle of protein-dye binding. *Anal Biochem* **72**: 248–254

Chang JY, Dethlefsen LA, Barley LR, Zhou BS and Cheng YC (1992) Characterization of camptothecin-resistant Chinese hamster lung cells. *Biochem Pharmacol* **43**: 2443–2452

Chen AY and Liu LF (1994) DNA topoisomerases: essential enzymes and lethal targets. *Annu Rev Pharmacol Toxicol* **34**: 191–218

Covey JM, D'Incalci M, Tilchen EJ, Zaharko DS and Kohn KW (1986) Differences in DNA damage produced by incorporation of 5-aza-2′-deoxycytidine or 5,6-dihydro-5-azacytidine into DNA of mammalian cells. *Cancer Res* **46**: 5511–5517

Dancey J and Eisenhauer EA (1996) Current perspectives on camptothecins in cancer treatment. *Br J Cancer* **74**: 327–338

de Leij L, Postmus PE, Buys CHCM, Elema JD, Ramaekers F, Poppema S, Brouwer M, van der Veen AY, Mesander G and Hauw TT (1985) Characterization of three new variant type cell lines derived from small cell carcinoma of the lung. *Cancer Res* **45**: 6024–6033

Deffie AM, Batra JK and Goldenberg GJ (1989) Direct correlation between DNA topoisomerase II activity and cytotoxicity in adriamycin-sensitive and -resistant P388 leukemia cell lines. *Cancer Res* **49**: 58–62

Eng WK, McCabe FL, Tan KB, Mattern MR, Hofmann GA, Woessner RD, Hertzberg RP and Johnson RK (1990) Development of a stable camptothecin-resistant subline of P388 leukemia with reduced topoisomerase I content. *Mol Pharmacol* **38**: 471–480

Fujimori A, Gupta M, Hoki Y and Pommier Y (1996) Acquired camptothecin resistance of human breast cancer MCF 7/C4 cells with normal topoisomerase I and elevated DNA repair. *Mol Pharmacol* **50**: 1472–1478

Goldwasser F, Bae I, Valenti M, Torres K and Pommier Y (1995) Topoisomerase I-related parameters and camptothecin activity in the colon carcinoma cell lines from the National Cancer Institute anticancer screen. *Cancer Res* **55**: 2116–2121

Goldwasser F, Shimizu T, Jackman J, Hoki Y, O'Connor PM, Kohn KW and Pommier Y (1996) Correlations between S and G2 arrest and the cytotoxicity of camptothecin in human colon carcinoma cells. *Cancer Res* **56**: 4430–4437

Holm C, Covey JM, Kerrigan D and Pommier Y (1989) Differential requirement of DNA replication for the cytotoxicity of DNA topoisomerase I and II inhibitors in Chinese hamster DC3F cells. *Cancer Res* **49**: 6365–6368

Hsiang YH, Lihou MG and Liu LF (1989) Arrest of replication forks by drug-stabilized topoisomerase I-DNA cleavable complexes as a mechanism of cell killing by camptothecin. *Cancer Res* **49**: 5077–5082

Jensen PB, Holm B, Sorensen M, Christensen IJ and Sehested M (1997) In vitro cross-resistance and collateral sensitivity in seven resistant small-cell lung cancer cell lines: preclinical identification of suitable drug partners to taxotere, taxol, topotecan and gemcitabin. *Br J Cancer* **75**: 869–877

Kataoka S, Naito M, Tomida A and Tsuruo T (1994) Resistance to antitumour agent-induced apoptosis in a mutant of human myeloid leukemia U937 cells. *Exp Cell Res* **215**: 199–205

Kohn KW (1981) DNA repair. In *A Manual of Research Techniques*, Friedberg EC and Hanawalt PC. (eds), pp. 379–401. Marcel Dekker: New York

Kohn KW (1991) Principles and practice of DNA filter elution. *Pharmacol Ther* **49**: 55–77

Miller SA, Dykes DD and Polesky HF (1988) A simple salting out procedure for extracting DNA from human nucleated cells. *Nucleic Acids Res* **16**: 1215

Nitiss J and Wang JC (1988) DNA topoisomerase-targeting antitumour drugs can be studied in yeast. *Proc Natl Acad Sci USA* **85**: 7501–7505

Ohashi N, Fujiwara Y, Yamaoka N, Katoh O, Satow Y and Yamakido M (1996) No alteration in DNA topoisomerase I gene related to CPT-11 resistance in human lung cancer. *Jpn J Cancer Res* **87**: 1280–1287

Pommier Y, Gupta M, Valenti M and Nieves-Neira W (1996) Cellular resistance to camptothecins. In *The Camptothecins. From Discovery to the Patient*, Pantazis P, Giovanella BC and Rothenberg ML. (eds), pp. 60–73. The New York Academy of Science: New York

Roed H, Christensen IJ, Vindelov LL, Spang Thomsen M and Hansen HH (1987) Inter-experiment variation and dependence on culture conditions in assaying the chemosensitivity of human small cell lung cancer cell lines. *Eur J Cancer Clin Oncol* **23**: 177–186

Rowinsky EK, Kaufmann SH, Baker SD, Miller CB, Sartorius SE, Bowling MK, Chen TL, Donehower RC and Gore SD (1996) A phase I and pharmacological study of topotecan infused over 30 minutes for five days in patients with refractory acute leukemia. *Clin Cancer Res* **2**: 1921–1930

Ryan AJ, Squires S, Strutt HL and Johnson RT (1991) Camptothecin cytotoxicity in mammalian cells is associated with the induction of persistent double strand breaks in replicating DNA. *Nucleic Acids Res* **19**: 3295–3300

Shimizu T, O'Connor PM, Kohn KW and Pommier Y (1995) Unscheduled activation of cyclin B1/Cdc2 kinase in human promyelocytic leukemia cell line HL60 cells undergoing apoptosis induced by DNA damage. *Cancer Res* **55**: 228–231

Slichenmyer WJ, Rowinsky EK, Grochow LB, Kaufmann SH and Donehower RC (1994) Camptothecin analogues: studies from the Johns Hopkins Oncology Center. *Cancer Chemother Pharmacol* **34**(suppl): S53–S57

Sorensen M, Sehested M and Jensen PB (1995) Characterisation of a human small-cell lung cancer cell line resistant to the DNA topoisomerase I-directed drug topotecan. *Br J Cancer* **72**: 399–404

 © Cancer Research Campaign 1998

Sugimoto Y, Tsukahara S, Oh hara T, Liu LF and Tsuruo T (1990) Elevated expression of DNA topoisomerase II in camptothecin-resistant human tumor cell lines. *Cancer Res* **50**: 7962–7965

Szmigiero L and Studzian K (1988) $H_2O_2$ as a DNA fragmenting agent in the alkaline elution interstrand crosslinking and DNA-protein crosslinking assays. *Anal Biochem* **168**: 88–93

Tamura H, Kohchi C, Yamada R, Ikeda T, Koiwai O, Patterson E, Keene JD, Okada K, Kjeldsen E and Nishikawa K (1991) Molecular cloning of a cDNA of a camptothecin-resistant human DNA topoisomerase I and identification of mutation sites. *Nucleic Acids Res* **19**: 69–75

Tobey RA (1972) Effects of cytosine arabinoside, daunomycin, mithramycin, azacytidine, adriamycin, and camptothecin on mammalian cell cycle traverse. *Cancer Res* **32**: 2720–2725

Tsao YP, D'Arpa P and Liu LF (1992) The involvement of active DNA synthesis in camptothecin-induced G2 arrest: altered regulation of p34cdc2/cyclin B. *Cancer Res* **52**: 1823–1829

Vindelov LL and Christensen IJ (1990) A review of techniques and results obtained in one laboratory by an integrated system of methods designed for routine clinical flow cytometric DNA analysis. *Cytometry* **11**: 753–770

© Cancer Research Campaign 1998